# **Quantum range-migration-algorithm for synthetic aperture **radar** applications**

**DOI:** 10.1038/s41598-023-38611-x

**Published:** 2023-07-15

**Authors:** Erik H. Waller, Andreas Keil, Fabian Friederich

**Affiliations:** 1 Fraunhofer-Institute for Industrial Mathematics ITWM, Fraunhofer-Platz 1, 67663 Kaiserslautern, Germany; 2 Becker Photonik GmbH, 32429 Minden, Germany

**Keywords:** Quantum simulation, Engineering

## Abstract

The 3D range-migration algorithm (RMA) and its 2D equivalent, the omega-k algorithm, are employed in a wide range of applications where reconstruction of synthetic aperture data is required, from satellite radar imaging of planets over seismic imaging of the earth crust, down to phased-array ultrasound and ultrasonic application, and recently in-line synthetic aperture radar for non-destructive testing. These algorithms are based on Fourier transforms and share their time-complexity. This limits highly-resolved measurement data to be processed at high speeds which would be advantageous for modern production feed lines. In this publication, we present the development and implementation of the RMA on a quantum computer that scales favourably compared to the time complexity of the classical RMA. We compare reconstruction results of simulated and measured data of the classical and quantum RMA. Hereby, the quantum RMA is run on a quantum simulator backend as well as on IBM’s Q System One quantum computer. The results show that real world applications and testing tasks may benefit from future quantum computers.

## Introduction

**Figure 1:**
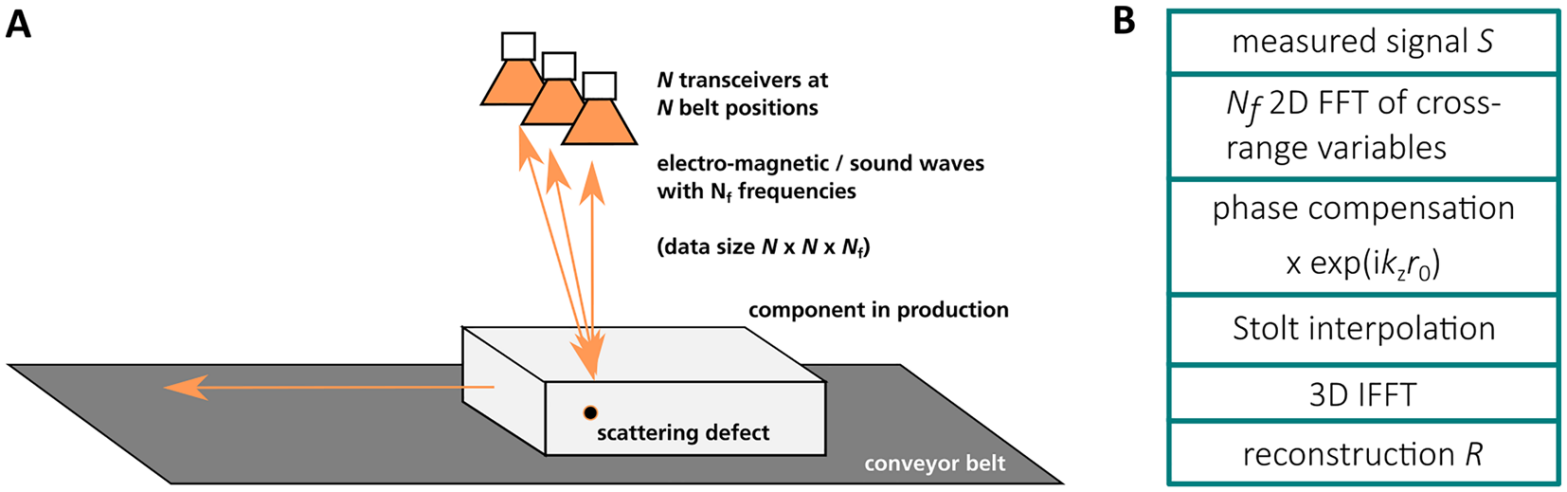
(**A**) Scheme of synthetic aperture radar measurement setup in an industrial non-destructive in-line testing scenario. (**B**) Scheme of the range migration algorithm*.*

Synthetic aperture radar (SAR) is a well-known measurement technique to obtain numerically focused volume information of a target volume from unfocused backscattered waves^1,2^. The physical nature of the waves hereby is not significant. E.g., in seismology and medical imaging often (ultra-)sound waves are used while in ground surveillance tasks often electro-magnetic (EM) radiation is employed. On the other hand, in industrial non-destructive testing mechanical soundwaves as well as microwave- and terahertz-frequency EM waves are exploited to probe optically non-transparent products for defects^3–5^. A typical industrial nondestructive testing scenario based on millimeter and terahertz waves is shown in Fig. 1A. A line array of transmit and receive antennas is used to illuminate the sample under test, which is moving on a conveyor. A general SAR imaging system would consist of an array of *N* transceivers that serially or simultaneously emit waves at *N*_f_ frequency points, measuring at N different conveyor-belt positions, giving cross-range and ground-range information, respectively. Being a lens-less system SAR requires sophisticated reconstruction algorithms (e.g., back-projection algorithm) that retrieve the scatterer positions and scattering strengths from the measured data. Among these reconstruction algorithms the range-migration algorithm (RMA, see Fig. 1B for a scheme) has the lowest complexity—especially when being implemented using fast Fourier transforms (FFTs)^6,7^. Still, the complexity of the 3D RMA scales with approximately 2*N*^2^*N*_f_ log_2_(*N*) + *N*^2^*N*_f_ + *N*^*2*^*N*_f_*M*_interp_ + *N*^2^*N*_f_log_2_(*N*^2^*N*_f_), corresponding to *N*_f_ 2D-FFTs, phase compensation, Stolt interpolation (*M*_interp_ being the complexity of the interpolation kernel) and the last term corresponding to the 3D inverse FFT^6^. Hereby, the highest computational costs stem from the Fourier transforms and depending on the interpolation kernel from the Stolt interpolation^8^. These high computational costs limit the application of highly resolved SAR imaging of large volumes for inline non-destructive testing using conventional CPUs or even GPUs. Contrary, the time complexity of the quantum Fourier transform (QFT) scales with log_2_(*N*)^2^ (2D-QFT: 2log_2_(*N*)^2^, 3D-QFT: 3log_2_(*N*)^2^)^9–12^. This scaling behavior of the RMA as a quantum algorithm compares favorably with the classical algorithm. However, while quantum algorithms exist and are well-known for the QFT, so far, the building blocks of the RMA, which is the fftshift operation, phase compensation and Stolt interpolation have not been published in literature and the respective computational costs are unknown. Here we present for the first time a full quantum RMA. The performance of the novel quantum RMA is compared to the classical RMA. Hereby, measured data is reconstructed using a quantum simulator backend and a classical computer, respectively. Furthermore, simulated data is reconstructed on a real quantum computer. We discuss the feasibility of the quantum RMA in the context of current and expected future limitations of quantum computers.

## Results

### Classical RMA

The principle of the measurement setup and the coordinate system is shown in Fig. 1A: at *N* conveyor-belt positions N transceivers emit waves at *N*_f_ frequencies. The waves are backscattered by a target and detected by the same transceiver. From this detected signal the scatterer positions and strengths need to be reconstructed by the RMA. The principle of the RMA is shown in Fig. 1B (for details see references^1^ and^8^): for each frequency a 2D FFT (including fftshift operations) of the cross-range variables is done, followed by a phase compensation which numerically refocuses the signal. Next, a Stolt interpolation flattens the curved wave fronts. Finally, a 3D inverse FFT transforms the data back to real space.

### Quantum RMA

An equivalent setup is to use one transceiver and move it to *N *× *N* different position to perform the measurement. For the measured signal we use the following signal model:

$$S\left( {x,y,{ }f} \right) = \mathop \sum \limits_{i} R_{i} exp\left( {2\pi j\frac{{2d_{i} \left( {x,y} \right)}}{c}f} \right)$$

where the image is considered to be a superposition of individual scatterers and *R*_i_ is the strength of the reflection, d the distance of the transceiver to that scatterer, c the velocity of light and f the frequency of the electromagnetic wave.

**Figure 2:**
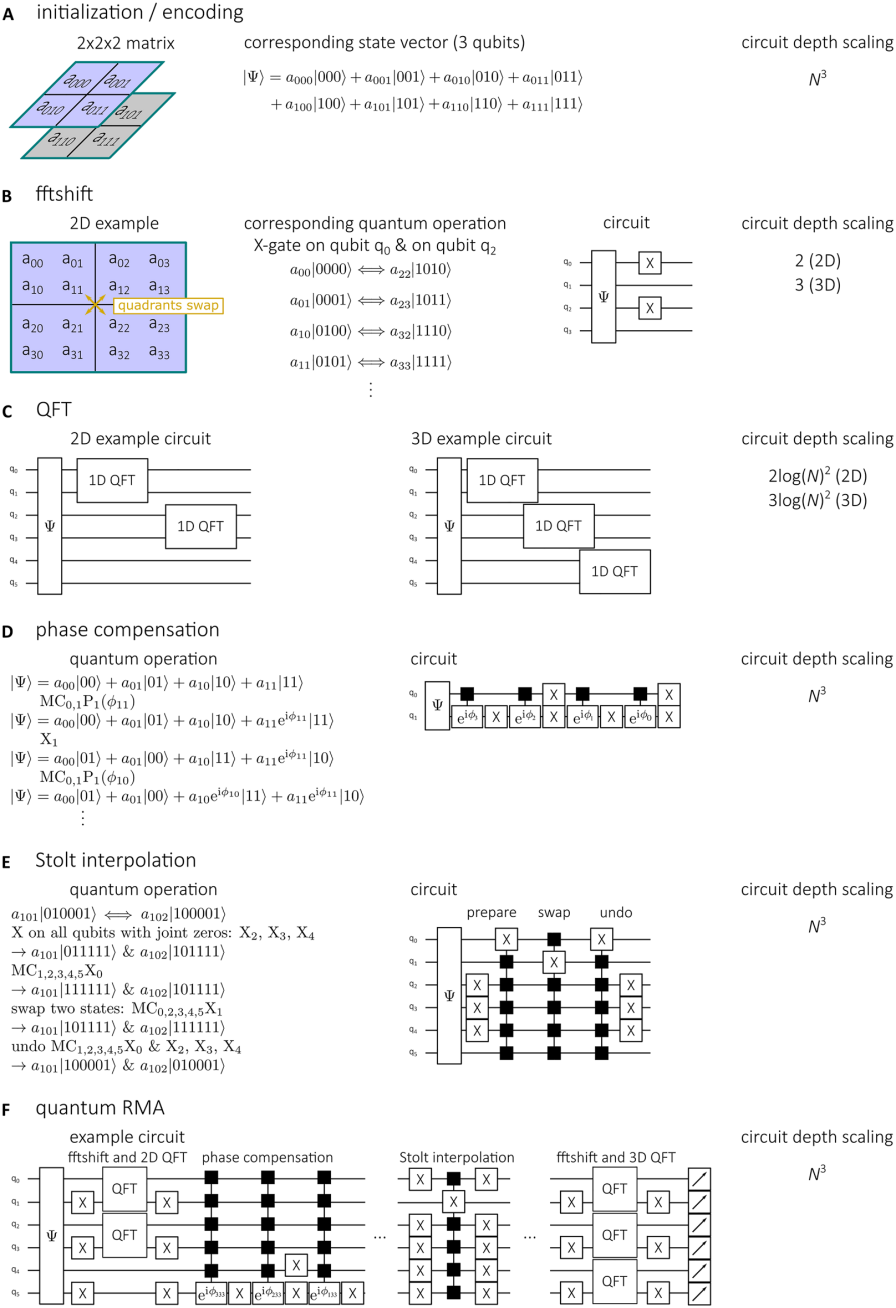
Subroutines of the quantum RMA: initialization (**A**), fftshift (**B**), 2D and 3D QFT (**C**), phase compensation (**D**), Stolt interpolation (**E**) and full quantum RMA (**F**). X denotes X-gates with the index corresponding to the qubit the gate is applied to. MC denotes multiple-controlled gates with the indices corresponding to the control qubits and P denotes the phase gate.

The complex measured signal *S(mΔx, nΔy, pΔf)* first needs to be pre-processed to enable quantum processing (*Δx and Δy* corresponding to the spatial sampling distances along *x* and *y* and *Δf* to the frequency sampling*)*. We encode the measured complex values *s*_mnp_ as the probability amplitudes *a*_mnp_ of a state vector (Fig. 2A). To this end, the values *s*_mnp_ are normalized to yield a total probability of one:

$$a_{mnp} = \frac{{s_{mnp} }}{{\mathop \sum \nolimits_{m = 0}^{N - 1} { }\mathop \sum \nolimits_{n = 0}^{N - 1} { }\mathop \sum \nolimits_{p = 0}^{{N_{f} - 1}} |s_{mnp} |^{2} }},\;\;{\text{which}}\;{\text{ensures}}\;\mathop \sum \limits_{m = 0}^{N - 1} \mathop \sum \limits_{n = 0}^{N - 1} \mathop \sum \limits_{p = 0}^{{N_{f} - 1}} |a_{mnp} |^{2} = 1.$$

The state vector is then initialized as:

$$\left| \Psi \right.\rangle = a_{000} \left| {0 \ldots 0} \right.\rangle + a_{001} \left| {0 \ldots 1} \right.\rangle + \cdots + a_{{N_{f} - 1,N - 1,N - 1}} \left| {1 \ldots 1} \right.\rangle$$

requiring 2log_2_(*N*) + log_2_(*N*_f_) qubits—e.g., for a dataset with 256 × 256 × 256 entries, 24 qubits. Note that this encoding scheme only gives relative amplitudes—therefore a dataset with all entries zero (a sample free of scatterers) and a dataset with all entries one (each probed sample point contains a scatterer) are indistinguishable^13^. In practice these two cases are, however, rarely encountered and therefore the chosen encoding scheme is well suited for the quantum RMA.

On the state vector a fftshift operation is performed. As in the classical case, this operation shifts the so called DC-term from the corners of an image to the centre, which is necessary to accurately correspond to a physical Fourier transform. This can, due to the chosen encoding, easily be done by X-gates on two qubits (2D) or three qubits (3D) (see Fig. 2B). Next, the 2D QFT over these cross-range variables is achieved by a 1D QFT over qubits that represent the *x* variable, followed by a 1D QFT over qubits that represent the *y* variable (Fig. 2C), followed by another quantum fftshift.

The next step, phase compensation, requires element-wise phase multiplication, meaning each *a*_mnp_ needs to be multiplied with a different phase factor. To this end, a multiple controlled phase multiplication is performed on the $a_{{N_{f} - 1,N - 1,N - 1}} |1 \ldots \left. 1 \right\rangle$ state to yield, $a_{{N_{f} - 1,N - 1,N - 1}} e^{{i\phi_{{N_{f} - 1,N - 1,N - 1}} }} |1 \ldots \left. 1 \right\rangle$. Next, the $|01\left. { \ldots 1} \right\rangle$ state is swapped with the $|11 \ldots \left. 1 \right\rangle$ state using an X-gate on the first qubit and then multiplied with the respective phase factor using controlled phase multiplication. This is continued until all basis states are multiplied with the respective phase factor (Fig. 2D).

Phase compensation is followed by Stolt interpolation which we implement as nearest neighbour interpolation. To determine which basis state is closest to the interpolation point the coordinates of the dataset in Fourier space are compared classically with an equidistant grid spanning from the minimum coordinate of the dataset to the maximum coordinate. Then swapping of the basis state with the basis state nearest to the interpolation point is performed. This makes it necessary to implement a routine that swaps two basis states only, leaving the other basis states unaltered. For this, the basis state that has more ones is prepared to the basis state that contains only ones using X- and multiple controlled X-gates. At the same time the other basis state is prepared to a state that contains only one zero. Then, a multiple controlled X-gate swaps those—and only those—two basis states. In the following, the steps needed for preparation of the two basis states are undone (Fig. 2E).

The final step of the quantum RMA is the 3D QFT which is composed of a quantum fftshift, 1D QFTs on the qubits representing the *x*-, *y*- and *z*-variable, respectively and another quantum fftshift.

The full quantum RMA circuit, including initialization and measurement operations, for a 4 × 4× 4 dataset is shown in Fig. 2F. Also refer to the supplementary material for more details.

### Quantum simulator backend

**Figure 3:**
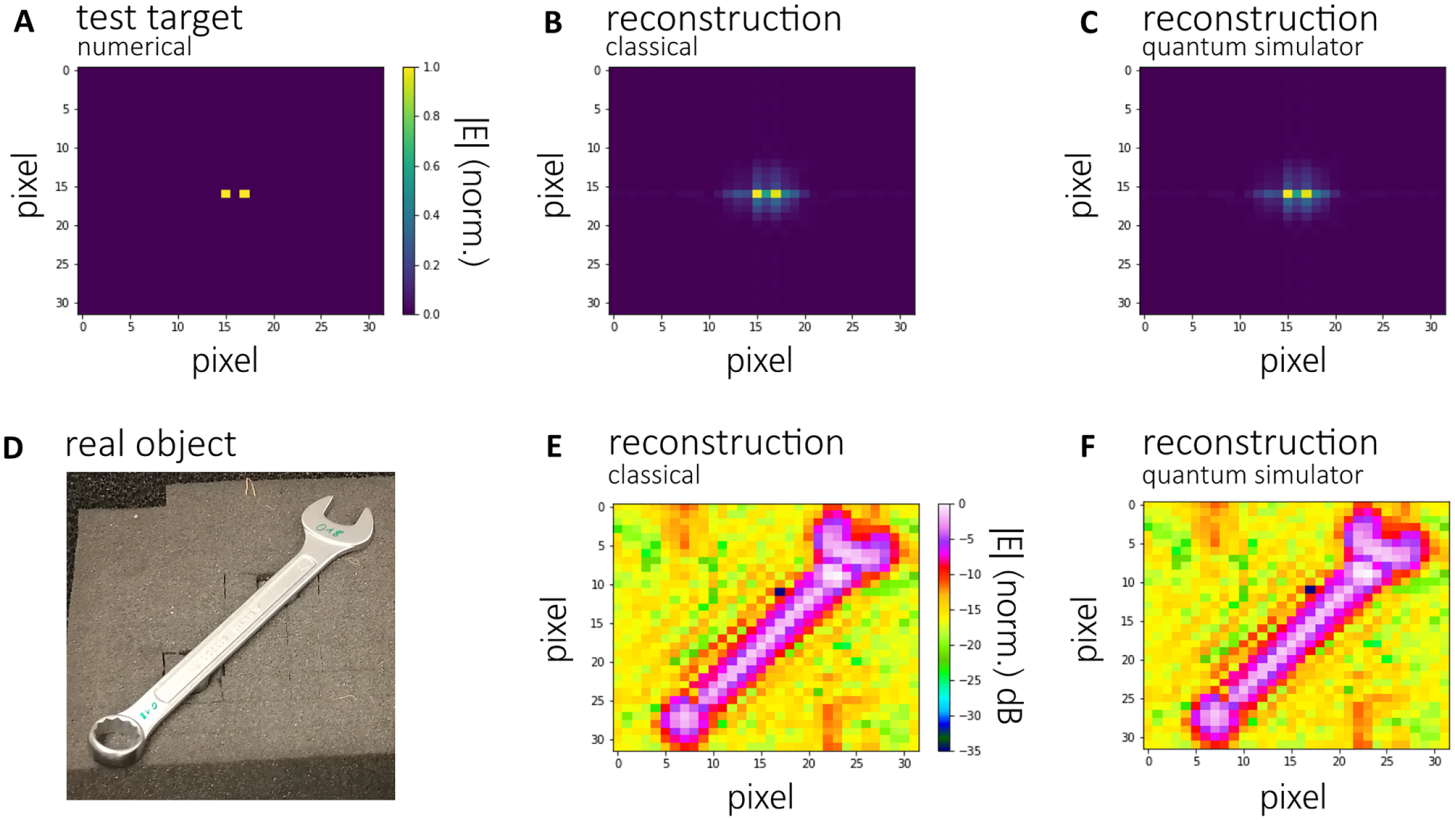
Numerically generated test pattern consisting of two scatterers (**A**) and reconstruction using classical RMA (**B**) and quantum RMA on a quantum simulator backend (**C**). Measurement of real object (**D**) and reconstruction using measured data: classical RMA (**E**), quantum RMA on simulator backend (**F**). While all calculations reconstructed a 3D volume only slices through this volume are shown for clarity.

To evaluate the proposed quantum RMA we calculated the signal generated by two scatterers 1 cm apart (Fig. 3A) inside a volume with 16 × 16 × 16 cm^3^ and detected by 32 × 32 transmitters (5 mm apart) emitting at 32 equidistant frequencies between 60 and 90 GHz at a working distance of 48 cm. This signal is reconstructed using the classical as well as the quantum RMA (the latter on IBM’s state vector simulator backend, see “Methods” section) with the reconstruction shown in Fig. 3B and C, respectively. We see that the reconstruction is identical for both algorithms.

To show the applicability of the quantum RMA in real world scenarios, we furthermore tested the algorithm against data measured by a terahertz scanner (see “Methods” for details of the scanner). Reconstructions of the object shown in Fig. 3D using classical RMA (Fig. 3E) and quantum RMA on the quantum simulator backend (Fig. 3F) both work well proving, that the quantum RMA is applicable for measurement tasks.

### Real quantum computer backend

**Figure 4:**
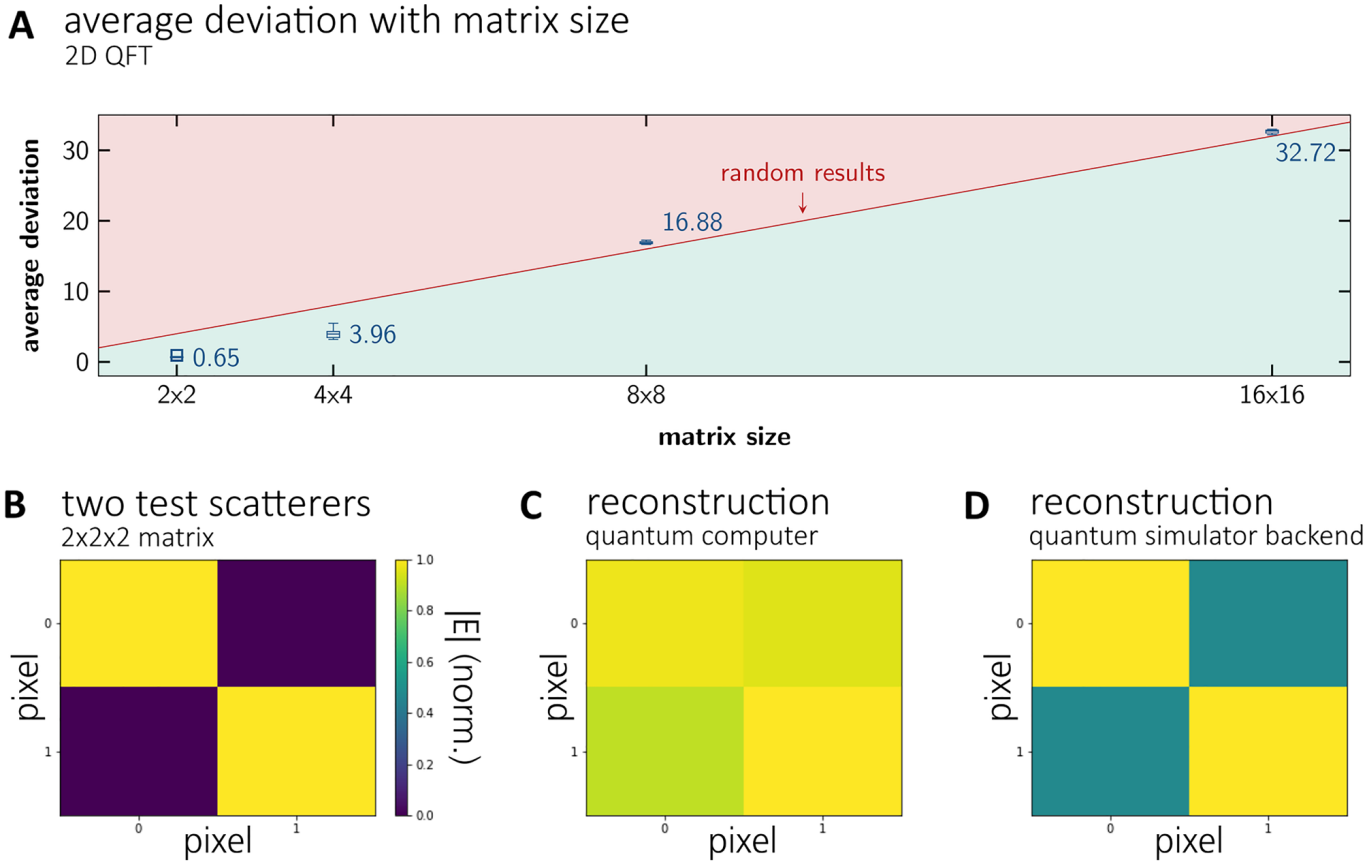
Boxplot of the average deviation between results of 2D classical FT and QFT depending on matrix size (**A**). The red line indicates the average deviation expected when all states are measured with equal probability. Numerically generated test scatterer (**B**) and reconstruction on a real quantum computer (**C**) as well as on a quantum simulator backend (**E**). While all calculations (**C**) and (**D**) reconstructed a 3D volume only slices through this volume are shown for clarity.

State of the art quantum computers still suffer from gate errors, rather short relaxation times and rather low cross-linking between qubits (low quantum volume). The low cross-linking between qubits requires many additional swap-operations to entangle non-cross-linked qubits, which increases the complexity of most quantum algorithms dramatically. Compared to classical algorithms this increase reduces the favourable complexity scaling behaviour of quantum algorithms with matrix size. Together with gate errors and limited relaxation times, calculations are prone to errors^13,14^. Since complexity of the algorithm increases with matrix size the total error also increases with it. In our experience, the most influential factors are CNOT gate errors and short relaxation times. To test the usability of IBM’s quantum computer for QFT based algorithms we first calculated 2D QFTs of test matrices (all entries with value two) of different size and compared the results with the results of classical 2D FFTs. No error correction was performed. In Fig. 4A the average deviation between those results is plotted as box plots (5 calculations on the quantum computer, 8192 shots each) against the matrix size. We see that starting from 8 × 8 matrix size, corresponding to 6 qubits, the results become essentially random, meaning we might as well have diced the result. Hereby, the random threshold is defined as the case when all states are measured with equal probability. This probability is then multiplied with a normalization factor necessary to enable quantitative comparison between classical and quantum results. Therefore, we restrict the performance test of our quantum RMA to a small data set of size 2 × 2 ×2, corresponding to 3 qubits. The reconstruction of two scatterers (Fig. 4B) is shown in Fig. 4C and compared to the reconstruction on a state vector simulator backend in Fig. 4D. The drop in signal-to-noise ratio of the reconstruction is clearly visible. Fortunately, this is not a fundamental issue but due to current quantum hardware limitations.

## Discussion and outlook

**Figure 5:**
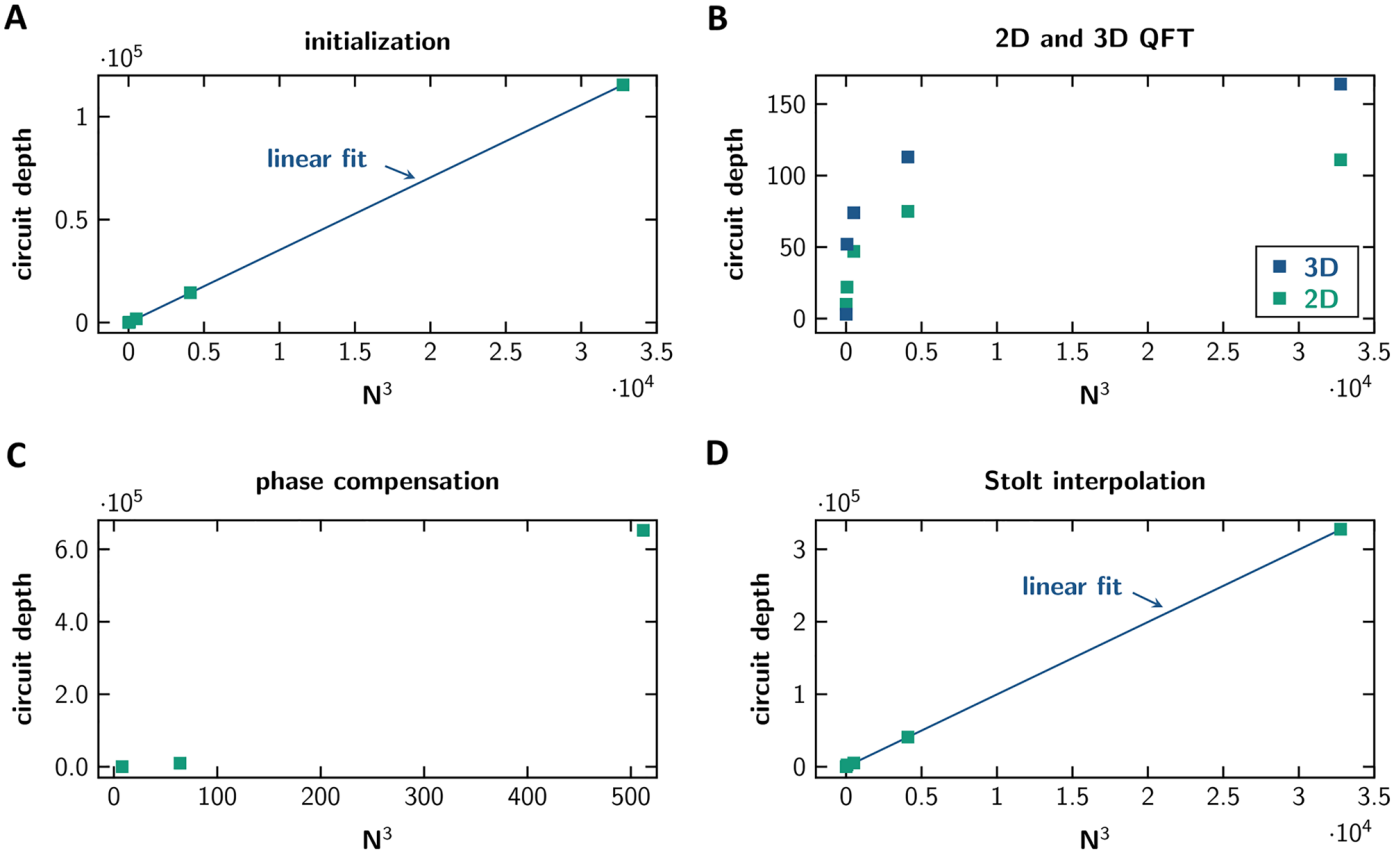
Circuit depth scaling vs. N^3^ for (**A**) initialization, (**B**) 2D and 3D QFT, (**C**) phase compensation and (**D**) Stolt interpolation. All routines are decomposed to basis gates.

It is worth noting that the most complex parts in classical algorithms may not coincide with the most complex parts in quantum algorithms. Therefore, we have a closer look at the complexity scaling behaviour of each subroutine of the quantum RMA. To this end the subroutines are run for datasets of different size on the state vector backend and circuit depth noted. Note, that here all gates are decomposed to a set of basis gates available to the real quantum computer. The scaling behaviour of these routines is presented in Fig. 5.

The first step of the quantum RMA is the initialization of the state vector. For the selected encoding scheme this routine’s complexity scales with *N*x*N*x*N*_f_. The complexity of the quantum fftshift is independent of matrix size but scales with the dimension of the matrix (2 for 2D quantum fftshift, 3 for 3D quantum fftshift). The 2D QFTs as previously mentioned scale with 2log_2_(*N*)^2^. Phase compensation complexity scales as initialization complexity with *N* ×*N* ×*N*_f_, however only, when future quantum computers provide efficient controlled phase gates (potentially directly implemented in hardware as single gates). If the controlled phase gate needs to be decomposed to basis gates, the complexity scaling gets much worse (see Fig. 5C). Contrary, with a smart choice of sampling parameters Stolt interpolation may be completely omitted when far-field conditions apply or subsampling in is acceptable. If needed, however, it also scales with *N*x*N*x*N*_f_. Finally, the 3D QFT scales with 2log_2_(*N*)^2^ + log_2_(*N*_f_)^2^.

In total, for large matrices each run of the quantum RMA scales at best with *N*^2^*N*_f_, if the above-mentioned controlled phase gates are available. Since each measurement only reads out one matrix entry (with a probability |*a*_*mnp*_|^2^), we need to run the algorithm at least SNR x #S times, where SNR is the desired signal-to-noise-ratio and #S the expected number of scatterers (or the maximum number of defects allowed in a product before being sorted out). These two values are usually independent of matrix size and therefore correspond to a factor so that the total complexity of the quantum RMA still scales with *N*^2^*N*_f_ (in the most general case where no a priori knowledge is available, e.g., in imaging either *N* runs need to be conducted, which destroys the quantum advantage, or quantum image recognition could be performed). Compared to the scaling of the classical RMA which scales at least with *N*^2^*N*_f_ log_2_(*N*) this is still an advantage of log_2_(*N*)—however only, if efficient controlled phase gates are available. If not, depending on the complexity of the controlled phase gates the classical RMA might even outperform the quantum RMA.

Clearly, initialization and especially phase compensation quantum routines need to be improved in future to further reduce the complexity of the quantum RMA. The first might either be achieved by future quantum measurement devices that directly make data available as an entangled state vector or by using phase-only data that is more efficiently initialized on a quantum computer. For the second, a routine that exploits radial symmetry of the phase multiplication factor may lead to complexity reduction. The image classification and interpretation should also be done on an quantum computer to reduce significantly the number of runs needed to extract the desired information. This is in the direction for future research on the problem of radar imaging with quantum computers. Together with improved future quantum computers the presented quantum RMA may efficiently be used in SAR applications.

## Methods

### Sampling criteria

For frequency sampling we used the common $\Delta f = \frac{c}{2H}$, where *H* is the height of the sample under test. The necessary bandwidth is determined by $B = \frac{c}{2\Delta y}$, where $\Delta y$ is the desired ground-range resolution. The necessary aperture *A* and its sampling $\Delta a$ is determined by the Fresnel sampling criteria: $A = \frac{{cr_{0} }}{{{\Delta }x f_{\max } }} - L$, where *L* is the length of the sample under test, $r_{0}$ the distance between aperture plane and centre of the object, $f_{max}$ the maximum frequency and $\Delta x$ the desired cross-range resolution.

### Quantum simulator backend

Quantum simulations were conducted using IBM’s state vector simulator backend^15^.

### Quantum computer calculation

The hardware backend we used is IBM’s quantum computer Ehningen with a Falcon processor^16^. Typical relaxation times were around 0.1 ms and typical CNOT errors around 10^–2^. Circuits were optimized by IBM’s own optimizer with optimization level 3. For all calculations we averaged 8192 shots.

### Data measurement

The measurements were performed in our lab with a single frequency-modulated continuous-wave (FMCW) transceiver, which is based on a pre-distorted voltage controlled oscillator (11.6–18.3 GHz) feeding both an active frequency multiplier chain emitter and a harmonic mixing receiver from Radiometer Physics GmbH. The emitter is an active frequency multiplier with a multiplication factor of 6 and the receiver a harmonic mixer at the 6th harmonic. Both are attached to a uni-directional coupler to provide a monostatic configuration in connection with a Pickett-Potter horn antenna. The transceiver is calibrated by using a metal plate as signal reference with perpendicular orientation to the measurement signal path in similar distance than the measurement sample. The transceiver was mechanically manipulated by linear translation stages to raster scan the target area. For each sweep 2000 frequency points of the receiver’s intermediate frequency output signal where acquired by a NI PCI-6115 data acquisition unit and downsampled to the described number of frequency points in the text.

## Supplementary Information

Supplementary Information.

## Data Availability

The datasets generated during and/or analysed during the current study are available from the corresponding author on reasonable request.
