# Supplementary material for: Quantum range-migration-algorithm for synthetic aperture radar applications
Source: Sci Rep. 2023 Jul 15;13:11436. doi: 10.1038/s41598-023-38611-x (PMC10349807; doi:10.1038/s41598-023-38611-x)
Supplement: Supplementary file 1 — Supplementary Information. [file 41598_2023_38611_MOESM1_ESM.docx]

Supplementary information to:

Quantum Range-Migration-Algorithm for Synthetic Aperture Radar Applications

­­­Erik H. Waller^1,*^, Andreas Keil^1,2^ & Fabian Friederich^1^

^1^ Fraunhofer-Institute for Industrial Mathematics ITWM, Fraunhofer-Platz 1, 67663 Kaiserslautern, Germany

^2^ Becker Photonik GmbH, 32429 Minden, Germany

^*^ Correspondence and requests for materials should be addressed to E. H. Waller (email: [erik.waller@itwm.fraunhofer.de](mailto:erik.waller@itwm.fraunhofer.de))

Mathematical background

The QRMA splits into different parts: First, the measured 3D matrix needs to be encoded into a normalized state vector. Then, the quantum analogues of an fftshift, 2D Fourier transforms and another fftshift follow. Next, quantum phase compensation and Stolt interpolation are performed. The algorithm finishes with another fftshift, a 3D Fourier transform, another fftshift and a measurement operation.

Initialization

Initialization of the state vector is achieved using Qiskits initialize class. This class uses different gates (e.g., Hadamard or Phase shift gates) to prepare the state vector. For clarity, we restrict ourselves to a 2D 4x4 matrix which requires 4 qubits encoding 16 matrix values:

$$a_{00} |0000> =a_{00} \left( \begin{aligned} 1 \\ 0 \end{aligned} \right)\otimes\left( \begin{aligned} 1 \\ 0 \end{aligned} \right)\otimes\left( \begin{aligned} 1 \\ 0 \end{aligned} \right)\otimes\left( \begin{aligned} 1 \\ 0 \end{aligned} \right)$$

$$a_{01} |0001> =a_{01} \left( \begin{aligned} 1 \\ 0 \end{aligned} \right)\otimes\left( \begin{aligned} 1 \\ 0 \end{aligned} \right)\otimes\left( \begin{aligned} 1 \\ 0 \end{aligned} \right)\otimes\left( \begin{aligned} 0 \\ 1 \end{aligned} \right)$$

and so on ($\otimes$ represents the tensor product). In total the state vector then reads:

$$|\psi> =\left( \begin{aligned} a_{00} \\ a_{01} \\ a_{02} \\ \vdots\\ a_{10} \\ a_{11} \\ \vdots\\ a_{33} \end{aligned} \right)$$

fftshift

The fftshift operation is achieved by an X-gate on qubit 0 and qubit 2:

$$a_{00} \left( \begin{matrix} 0 & 1 \\ 1 & 0 \end{matrix} \right)\left( \begin{aligned} 1 \\ 0 \end{aligned} \right)\otimes\left( \begin{aligned} 1 \\ 0 \end{aligned} \right)\otimes\left( \begin{matrix} 0 & 1 \\ 1 & 0 \end{matrix} \right)\left( \begin{aligned} 1 \\ 0 \end{aligned} \right)\otimes\left( \begin{aligned} 1 \\ 0 \end{aligned} \right)= a_{00} |1010>$$

$$a_{01} \left( \begin{matrix} 0 & 1 \\ 1 & 0 \end{matrix} \right)\left( \begin{aligned} 1 \\ 0 \end{aligned} \right)\otimes\left( \begin{aligned} 1 \\ 0 \end{aligned} \right)\otimes\left( \begin{matrix} 0 & 1 \\ 1 & 0 \end{matrix} \right)\left( \begin{aligned} 1 \\ 0 \end{aligned} \right)\otimes\left( \begin{aligned} 0 \\ 1 \end{aligned} \right)= a_{01} |1011>$$

and so on ($\left( \begin{matrix} 0 & 1 \\ 1 & 0 \end{matrix} \right)$ corresponds to the X-gate). Effectively, this results rearrangement of the state vector which corresponds to a quadrant swap in the classical 2D matrix picture. The latter, in turn, is exactly what the classical fftshift does.

2D Fourier transformation

The 1D quantum Fourier transformation has extensively been covered (see references in the manuscript) and will therefore omitted here. The 2D version and 3D version are just an extension to the 1D version:

$$QFT\{a_{00} \left( \begin{aligned} 1 \\ 0 \end{aligned} \right)\otimes\left( \begin{aligned} 1 \\ 0 \end{aligned} \right)\}\otimes QFT\{\left( \begin{aligned} 1 \\ 0 \end{aligned} \right)\otimes\left( \begin{aligned} 1 \\ 0 \end{aligned} \right)\}$$

$$QFT\{a_{01} \left( \begin{aligned} 1 \\ 0 \end{aligned} \right)\otimes\left( \begin{aligned} 1 \\ 0 \end{aligned} \right)\}\otimes QFT\{\left( \begin{aligned} 1 \\ 0 \end{aligned} \right)\otimes\left( \begin{aligned} 0 \\ 1 \end{aligned} \right)\}$$

and so on (QFT here is an abbreviation of the 1D QFT operation).

Phase compensation

In this step, all matrix entries are multiplied by a different phase value. Here, we use multiple controlled phase gates and X-gates to achieve this goal. First, the respective phase is multiplied only to the |1111> state using the multiple controlled phase gate:

$$\left( \begin{matrix} 1 & 0 & \cdots& 0 \\ 0 & 1 & \ldots& 0 \\ \vdots& \vdots& \ddots& \vdots\\ 0 & 0 & \cdots& e^{i\phi_{33}} \end{matrix} \right)\left( \begin{matrix} a_{00} \\ a_{01} \\ \vdots\\ a_{33} \end{matrix} \right)=\left( \begin{matrix} a_{00} \\ a_{01} \\ \vdots\\ a_{33}\times e^{i\phi_{33}} \end{matrix} \right)$$

Then application of an X-gate on the last qubit swaps the |1111> state with the |1110> state but also swaps other states, e.g.:

$$a_{31} \left( \begin{aligned} 0 \\ 1 \end{aligned} \right)\otimes\left( \begin{aligned} 0 \\ 1 \end{aligned} \right)\otimes\left( \begin{aligned} 1 \\ 0 \end{aligned} \right)\otimes\left( \begin{matrix} 0 & 1 \\ 1 & 0 \end{matrix} \right)\left( \begin{aligned} 0 \\ 1 \end{aligned} \right)= a_{31} |1100>$$

$$a_{32} \left( \begin{aligned} 0 \\ 1 \end{aligned} \right)\otimes\left( \begin{aligned} 0 \\ 1 \end{aligned} \right)\otimes\left( \begin{aligned} 0 \\ 1 \end{aligned} \right)\otimes\left( \begin{matrix} 0 & 1 \\ 1 & 0 \end{matrix} \right)\left( \begin{aligned} 1 \\ 0 \end{aligned} \right)= a_{32} |1111>$$

$$a_{33} \times e^{i\phi_{33}} \left( \begin{aligned} 0 \\ 1 \end{aligned} \right)\otimes\left( \begin{aligned} 0 \\ 1 \end{aligned} \right)\otimes\left( \begin{aligned} 0 \\ 1 \end{aligned} \right)\otimes\left( \begin{matrix} 0 & 1 \\ 1 & 0 \end{matrix} \right)\left( \begin{aligned} 0 \\ 1 \end{aligned} \right)= a_{33} \times e^{i\phi_{33}} |1110>$$

Then, again a multiple controlled phase gate is applied:

$$\left( \begin{matrix} 1 & 0 & \cdots& 0 \\ 0 & \ddots& \ldots& 0 \\ \vdots& \vdots& e^{i\phi_{32}} & \vdots\\ 0 & 0 & \cdots& 1 \end{matrix} \right)\left( \begin{matrix} a_{00} \\ \vdots\\ a_{32} \\ a_{33}\times e^{i\phi_{33}} \end{matrix} \right)=\left( \begin{matrix} a_{00} \\ \vdots\\ a_{32} \times e^{i\phi_{32}} \\ a_{33}\times e^{i\phi_{33}} \end{matrix} \right)$$

Then, two X-gates need to be applied to turn a_31_|1100> to a_31_|1111> and so on. This scheme is followed until all states have been multiplied with their phase value.

Stolt interpolation

Interpolation is here achieved by swapping a basis state with the basis state nearest to the interpolation point. To this end, X-gates and multiple controlled X-gates are used. E.g., when states |1111> and |1110> need to be swapped, a multiple controlled X-gate with the control being q_0_, q_1_ and q_2_ while q_3_ is the target does the trick. When, e.g., |1000> and |1100> need to be swapped, more preparation needs to be done. We first prepare the state with more ones into a state with only ones using X-gates on q_2_ and q_3_. This will change the two above states to |1011> and |1111>, respectively, however, also swaps all other basis states. In the next step only those two states are swapped using controlled X-gates with the control on q_0_, q_2_, q_3_ and the target on q_1_, yielding |1111> and |1011>, respectively. Since all other basis states have also been swapped in the preparation step, this effect needs to be undone. This can be achieved by simply applying the X-gates on q_2_ and q_3_ again. This yields |1100> and |1000> for the two states that needed to be swapped and leaves all other basis states unaltered.

3D Fourier transformation

The 3D QFT follows the same scheme as the 2D QFT.

Measurement

The measurement is the multiplication of the state vector with its complex conjugate. The absolute squared of the complex amplitudes of each basis state represent the probabilities with which the corresponding basis state is measured. For the paper’s use-case of imaging, only the amplitudes are utilized and we do not need to employ any phase retrieval algorithm.
